# The Atopic March: Progression from Atopic Dermatitis to Allergic Rhinitis and Asthma

**DOI:** 10.4172/2155-9899.1000202

**Published:** 2014-04-07

**Authors:** Selene K. Bantz, Zhou Zhu, Tao Zheng

**Affiliations:** Section of Allergy and Clinical Immunology, Yale University, School of Medicine, New Haven, 06520, USA

**Keywords:** Eczema, Atopic dermatitis, Allergic rhinitis, Asthma, The atopic march

## Abstract

The development of atopic dermatitis (AD) in infancy and subsequent allergic rhinitis and asthma in later childhood is known as the atopic march. This progressive atopy is dependent on various underlying factors such as the presence of filaggrin mutations as well as the time of onset and severity of AD. Clinical manifestations vary among individuals. Previously it was thought that atopic disorders may be unrelated with sequential development. Recent studies support the idea of a causal link between AD and later onset atopic disorders. These studies suggest that a dysfunctional skin barrier serves as a site for allergic sensitization to antigens and colonization of bacterial super antigens. This induces systemic Th2 immunity that predisposes patients to allergic nasal responses and promotes airway hyper reactivity. While AD often starts early in life and is a chronic condition, new research signifies that there may be an optimal window of time in which targeting the skin barrier with therapeutic interventions may prevent subsequent atopic disorders. In this review we highlight recent studies describing factors important in the development of atopic disorders and new insights in our understanding of the pathogenesis of the atopic march.

## Introduction

Atopic diseases, including atopic dermatitis, allergic rhinitis, and asthma have increased in frequency in recent decades and now affect approximately 20% of the population worldwide. The concept of the atopic march was developed to describe the progression of atopic disorders from atopic dermatitis (AD) in infants to allergic rhinitis and asthma in children [1]. Patients with AD may develop a typical sequence of AD, allergic rhinitis and asthma at certain ages. Some may have disease that persists for several years, whereas others may see improvement or resolution with increasing age [2]. Atopy is defined as a personal or familial propensity to produce IgE antibodies and sensitization in response to environmental triggers [3]. Underlying atopy has been considered to be critical in linking AD, allergic rhinitis and asthma [1,4]. The risk of developing atopic diseases is complex and the temporal pattern described in the atopic march may not be a simple progression. The development of these diseases is strongly influenced by both genetic and environmental factors. While these disorders share risk factors, the nature and development of disease can vary among individuals. Atopic diseases can be unrelated disorders that develop sequentially along an atopic pathway or there may be a causal link between eczema and the later-onset atopic respiratory disorders. However, the concept of the atopic march has been supported by cross-sectional and longitudinal studies [5–14] and is further confirmed when examining data on the prevalence of each atopic disease across population life spans as well as by experimental evidence from mouse models.

## The First Step of the Atopic March: Atopic Dermatitis

Many studies refer specifically to AD; in this review the terms atopic dermatitis and eczema are considered interchangeable. Atopic dermatitis is a chronic pruritic skin disease. In the International Study of Asthma and Allergies in Childhood (ISAAC), the prevalence of AD in children varies significantly from 0.3% to 20.5% among 56 countries, but there are consistent trends of increasing disease prevalence over time [15,16]. A Polish study determined the prevalence of atopic dermatitis in infants less than 6 months old to be 17.3% [17], and an international study found a similar atopic dermatitis prevalence of 17.6% in children aged 1–2 years [18]. A population-based study in the US illustrates that AD starts early in the first few years of life. Of affected children aged 3–11 years, 85% suffered from AD before 5 years of age, including 45% who developed the condition during the first 6 months of life and 60% who developed the condition during first year of life [4,19]. Less than half of patients with AD have complete resolution by 7 years of age and only 60% of them have resolution by adulthood, indicating the chronic nature of AD [1,4,20]. An important aspect in the natural history of AD is whether patients will outgrow their disease, and this is discussed in several articles detailed below. The mechanism of how to "outgrow" AD remains largely unknown and may be influenced by both genetic and environmental factors [12].

The mainstays of therapy for AD include frequent moisturizing barrier cream, optimal skin hygiene, avoidance of allergic triggers, topical corticosteroids, and calcineurin inhibitors. Antihistamines can help control itch. Emollients improve the barrier function of the stratum corneum by providing water and lipids. Sufficient lipid replacement therapy reduces the inflammation and restores epidermal function. Some products have therapeutic efficacy in improving clinical and biophysical parameters of patients with AD. Long-term studies would be important to evaluate whether lipid barrier replacement therapy reduces bacterial colonization or prevents progression of the atopic march [21].

## The End Points (Progression) of the Atopic March: Allergic Rhinitis and Asthma

AD is a major risk factor for the development of asthma, and children with AD have an increased odds ratio of developing asthma compared to children without AD in several longitudinal studies. Patients with eczema with specific IgE antibodies to common environmental allergens (extrinsic AD) present by 2 to 4 years of age are at higher risk for progressing in the atopic march to allergic rhinitis and asthma than those with eczema without IgE sensitization (intrinsic AD) [10,22]. The main risk factors for progression and persistence of asthma are IgE sensitization and early onset and severity of AD. The estimated odds risk for the association of eczema at 2 years with asthma at 6 years is about 1.80 [23]. Additionally, approximately 70% of patients with severe AD develop asthma compared to 20–30% of patients with mild AD and approximately 8% of the general population. The severity of AD correlates with the risk of developing rhinitis and with elevated levels of total and specific IgE antibodies [6].

AD is highlighted as the first step of the atopic march in numerous cross-sectional and longitudinal studies. One review by von Kobyletzki found that children with eczema have 3-fold increased odds of developing asthma and nearly 3-fold increased odds of developing rhinitis at 5-year follow up compared to children without eczema. When eczema is further subdivided, having severe eczema, early-onset eczema, and persistent eczema further increases the risks of developing asthma and rhinitis [18]. A prospective, population-based study found that despite most cases of eczema being mild to moderate, the co-existence of different allergy-related diseases of eczema, asthma, and allergic rhinoconjunctivitis at 6 years is higher among those with the onset of eczema before 2 years of age [23]. About 1 in every 3 children with eczema develops asthma during later childhood [13]. Another study [24] showed that children with infantile eczema have a 3-fold risk of having eczema in preadolescence compared to children without eczema before age 2 years. Infantile eczema was defined by either a physician diagnosis or parental report of dry, itchy rashes for at least 2 weeks, occurring between ages 3 months and 2 years in a typical location such as the face, extensor or flexural surfaces of arms and legs, or flexural surfaces of wrists and ankles. The same study demonstrated that children with infantile eczema have an increased risk of asthma and rhinitis in preadolescence that cannot be attributed to preadolescent eczema with concomitant asthma and rhinitis. This was shown by analyses which restricted those children with preadolescent eczema and found that among children who did not have eczema in preadolescence, children with a history of infantile eczema still have an increased risk of asthma and rhinitis in preadolescence compared to their peers. This is auspicious for an existing atopic march from early eczema to asthma and rhinitis. Almost half of children with infantile eczema will have eczema, asthma, or rhinitis in preadolescence [24].

The Tasmanian Longitudinal Health (TLH) Study investigated the influence of eczema on the development of asthma from childhood to adult life and found that childhood eczema was significantly associated with new-onset asthma in three separate life stages: pre-adolescence (hazard ratio 1.70; 95% confidence interval [CI] 1.05–2.75), adolescence (2.14; 1.33–3.46), and adult life (1.63; 1.28–2.09) as well as over the life-span from the ages of 8 to 44 years (1.73; 1.42–2.12) [25]. One review by van der Hulst et al. [13] examined 13 prospective extrinsic AD cohort studies and found the prevalence of asthma in AD cohorts at the age of 6 to be about 30%. Kapoor et al. [7] examined the prevalence of allergic rhinitis and asthma in 2,270 children with physician-confirmed AD and found that by 3 years of age, nearly 66% of the subjects reported to have allergic rhinitis, asthma, or both, and the presence of these diseases correlated with poor AD control. A population-based prevalence estimate of eczema in U.S. adults found that the 1 year eczema prevalence of adults is 10.2%, with 3.2% of the adult population ever having a history of asthma, hay fever, or both [26]. These studies strongly suggest that the atopic march progresses well past childhood. It is still unclear why some infants with AD outgrow the disease with increasing age, whereas others will "march" to develop other atopic conditions such as allergic rhinitis and/or asthma in later stages.

Epidemiologic studies illustrate strong associations between rhinitis and asthma [27–31]. Allergic rhinitis is an inflammatory condition affecting nasal mucosal membranes. In sensitized individuals, allergens such as pollens, molds, and animal dander provoke this allergic response. Although allergic rhinitis is often trivialized, it has a significant impact on quality of life and substantial socioeconomic consequences, and it is associated with multiple comorbidities, including asthma. Cardinal features of asthma include airway inflammation and airway hyperreactivity to allergens, associated with structural remodeling. Studies on the prevalence of asthma in patients with rhinitis vary considerably, but it has been reported to be as high as 80% [32]. Many patients with allergic rhinitis have lower airway hyperreactivity or bronchial hyperresponsiveness. Allergic rhinitis as a risk factor for developing asthma has been supported by several studies [4,33]. Ciprandi et al. [33] showed that nasal symptoms, airflow, and markers of inflammation (eosinophils, Th2 cytokine levels) directly correlate with lower airway markers including forced expiratory volume in 1 second (FEV1). Leynaert et al. [30] found that approximately 75% of subjects with asthma report rhinitis; patients with rhinitis have increased risk for asthma and lower airway reactivity compared to patients without rhinitis; and the risk for asthma increases from 2.0% in subjects without rhinitis to 18.8% in subjects with allergic rhinitis when exposed to either pollen or animal dander. These studies suggest that allergic rhinitis is a risk factor for asthma and can precede asthma in the atopic march.

A prospective 10-year follow-up study investigated whether the atopic march theory could be applied to local allergic rhinitis, a newly described entity in which patients demonstrate elevated local specific IgE and markers of allergic inflammation with positive nasal provocation tests but exhibit no systemic sensitization with negative skin prick testing and serum specific IgE levels. However, the study found similar rates of development of systemic atopy in patients with local allergic rhinitis and healthy controls, suggesting that local allergic rhinitis is an entity well differentiated from allergic rhinitis, and not a clear step in the atopic march. Therefore, the prevailing theory remains that atopic dermatitis defines the initial step of the atopic march, and the main pathway of early allergic sensitization occurs through the skin in patients with atopic dermatitis [34]. One study [35] based on the TLH Study examined the role of eczema on hay fever from a familial perspective. By using a regression model that accounted for familial predictors, the study could test whether data was consistent with the existence of a causal component by examining whether the association of an outcome with the predictor of a sibling was attenuated after adjusting for self predictor status. The study concluded that the association of infantile eczema on asthma in children without hay fever is unlikely to be causal or familial, but rather the result of individual-specific covariates such as respiratory tract infections. The study also showed that the contribution of eczema to hay fever and asthma in childhood varies for different phenotypes and is unlikely to be completely explained by familial confounding. This suggested that infantile eczema is causal for some hay fever in childhood, especially in asthmatic children (about 30%), compared to about 10% of hay fever in children without asthma. This provides further credence that it might be possible to prevent hay fever in children with eczema by controlling their eczema and improving skin barrier function [35].

## Role of Food Allergy in the Atopic March

Over the past decade, a significant increase in the prevalence of food allergy-related anaphylaxis [36,37] indicates that there is a rise in food allergy. Atopic dermatitis and food allergy commonly co-exist, particularly in those with early onset, severe and persistent atopic eczema. Food allergy is a known provoking cause of AD and the prevalence of IgE-mediated food allergy is about 35% in children affected with AD [38]. Whether children with IgE-mediated food allergy are at increased risk of developing subsequent other allergic manifestations such as asthma and allergic rhinitis is unclear. One study found that early sensitization to food and the presence of a filaggrin mutation in infants with early-onset eczema each increased the risk for persistent eczema and for subsequent asthma, although the combination of the 2 factors had low sensitivity in reliably identifying children at risk [39]. In another study, investigators prospectively followed 118 children with cow's milk allergy (CMA) at baseline and assessed whether challenge-proven CMA in infancy predisposes children to bronchial hyperresponsiveness at school age. They found that children with a history of IgE-positive CMA diagnosed at a mean age of 7 months, not IgE-negative CMA, exhibited increased airway inflammation and higher bronchial responsiveness to histamine at 8 years of age [40,41]. It is unclear whether the progression from IgE-mediated food allergy to asthma in subjects without eczema is causal or a result of shared environment and/or shared genetics. Because eczema and food allergy can co-exist in infants, it is also unclear whether the observed association is related to co-manifestation of other allergic conditions such as eczema and allergic rhinitis that predict asthma or if it is a consequence of food allergy itself. It is important to have large population-based prospective cohorts to include food allergy as a baseline outcome to further investigate whether food allergy truly represents an initial step of the atopic march in infants with shared environmental and genetic determinants or whether it is an independent predictor.

## Animal Models Supporting the Atopic March

Environmental and genetic studies provide evidence that a defect in epithelial barrier integrity may contribute to the onset of AD and progression of the atopic march. Many studies in animal models demonstrate that epidermal barrier dysfunction can be caused by repeated sensitization to allergens to the skin, which leads to phenotypes of AD systemic sensitization and increased risk of allergic rhinitis lung inflammation and airway hyperresponsiveness [42,43]. A study in a mouse model showed that epicutaneous aeroallergen exposure induces systemic Th2 immunity that predisposes to allergic nasal responses, suggesting that the skin is a potent site for antigen sensitization in the development of experimental allergic rhinitis [44]. In addition, the progression from AD to asthma in mice is supported by the data that epicutaneous sensitization with ovalbumin induces localized AD and airway hyperresponsiveness to methacholine after challenge with aerosolized ovalbumin [43]. Indeed, murine models have shown that epicutaneous exposure to ovalbumin and peanut after the removal of the stratum corneum induces a strong systemic Th2 immune responses characterized by elevated IL-4 secretion by T cells from draining lymph nodes and high levels of allergen specific IgE and IgG1 [38,45]. Thymic stromal lymphopoietin (TSLP) in the pathogenesis in human AD has been well documented, and TSLP is shown to be highly increased in skin and blood of patients with AD [46,47]. However, its role in the atopic march in humans remains to be defined. We and others show that the expression of TSLP is strongly increased by keratinocytes of AD skin in IL-13 transgenic mice by immunohistochemistry and ELISA [48] and that topical application of vitamin D3 induces TSLP expression in mouse keratinocytes and triggers AD [49]. TSLP, when overexpressed by skin keratinocytes, is a systemic driver of bronchial hyperresponsiveness and its deletion prevents the atopic march from occurring, suggesting that keratinocyte-produced TSLP may be involved in the link of AD to asthma [50]. Trefoil Factor 2 is another mediator with important functions in epithelial barrier function and repair that rapidly induces IL-33 during allergic asthma. IL-33 is an alarmin and a damage-associated molecular pattern that promotes both Th2 and protective antiviral CD8+ T-cell responses [51]. A possible role of IL-17 in the atopic march is supported by a study showing that ovalbumin inhalation by epicutaneously-sensitized mice induced expression of IL-17 and bronchial hyperreactivity, which are reversed by IL-17 blockade [42]. Patients with AD have a unique predisposition to colonization or infection by *Staphyloccous aureus*. About 70% of isolated *S. aureus* produce bacterial exotoxins with superantigen (SAg) properties and there is a positive correlation between AD severity and staphylococcal SAgs, including staphylococcal enterotoxin B (SEB) [52–58]. We and others showed in murine models of AD that when combined with allergens, SEB has an additive and synergistic effect on driving cutaneous eczematoid skin changes [59,60] and promotes airway hyperreactivity and lung inflammation upon allergen challenge [59].

## Potential Mechanisms and Speculations Underlying the Atopic March

Previous approaches to understanding AD have centered on mechanisms in the adaptive immune system, often with an emphasis on the Th1-Th2 paradigm. The conceptual focus has been increasingly shifting to include a primary defect in the epithelial barrier as a threshold event in AD. The epidermis provides an essential attribute to the integrity of the occlusive interface barrier, restricting both water loss from the body and ingress of pathogens. This barrier is formed after complex and integrated biochemical events. Epithelial keratinocytes replace their plasma membrane with a tough, insoluble layer termed the cornified envelope to achieve and maintain this barrier to prevent infectious agents and allergens from gaining access to the body. The lack of dermal integrity is clearly an important part that begins allergic sensitization in the atopic march. Another theory is that lack of exposure to microorganisms helps facilitate an allergic phenotype. Toll-like receptors link the atopic march to the hygiene hypothesis, as dermal exposure to lipopolysaccharide during allergen sensitization modulates the asthmatic response by skewing the Th1/Th2 balance toward Th1 by stimulating the production of IFN-γ. These findings support the hygiene hypothesis and pinpoint the importance of the dermal microbiome in the development of allergy and asthma [61].

Although it has become evident that the mechanisms by which allergen exposure occurs through impaired skin barriers can initiate systemic allergy and predispose individuals to AD, allergic rhinitis, and asthma, the cause of AD remains incompletely understood, and the mechanisms of the atopic march are still largely unknown.

## Skin Barrier Defects in AD and the Atopic March

The epidermis functions as a primary defense and biosensor to the external environment. Skin barrier defects promote easy entry for pathogens, allergens and other environmental insults such as toxins, irritants, pollutants and are now considered the primary mechanism of development of AD [62]. The skin barrier function is impaired in AD as a consequence of multiple abnormalities responsible for the barrier defect including reduced lipids (ceramide and sphingosine) and abnormal keratinization due to dysfunctional filaggrin, a critical component in the cornified envelope formation [63–68]. Clinically the disrupted skin barrier is supported by the increased transepidermal water loss (TEWL) observed in both lesional and nonlesional skin [62,69,70]. Increased TEWL correlates with increased AD severity [71]. AD keratinocytes have an aberrant response to environmental triggers and are able to produce a unique profile of cytokines including IL-13, TSLP, and chemokines that promote Th2 predominant inflammatory responses in acute AD lesions followed by chronic AD characterized by prominent Th1 inflammation [72]. IL-13 has been found to induce AD and the atopic march via a TSLP-dependent mechanism [73]. Studies have also demonstrated exaggerated expression of IL-13 and IL-22 in both acute and chronic lesions of patients with AD [74]. The importance of the role of IL-22 in human AD has become clearer as recent studies have demonstrated the frequency of IL-22–producing T cells in AD skin positively correlates with disease severity and the newly identified IL-13/IL-22–coproducing CD4^+^ T cells carrying cutaneous lymphocyte antigen contribute to the pathogenesis of AD [75–77]. Both Th2 and Th22 cytokines inhibit epidermal differentiation and thereby contribute to the reduced filaggrin expression and anti-microbial peptide production which leads to increased susceptibility to *S. aureus* colonization in AD patients [78–81]. The impaired skin barrier is often further compromised by chronic heavy colonization of *Staphylococcus aureus*, which occurs in 90% of AD patients [82]. Superantigens secreted from *S. aureus* in AD skin further stimulate keratinocytes to produce TSLP and induce polyclonal activation of T cells via binding directly to the common variable β (vβ) chains of T-cell receptors [83–85], which results in exaggerated Th2 inflammatory responses leading to worsening AD, and this can also promote systemic Th2 responses and allergic lung inflammation through an IL-17A dependent mechanism [59]. Respiratory infections such as RSV bronchiolitis can also predispose to wheeze, and because prophylaxis with pavlizumab is associated with reduced frequency of subsequent childhood wheezing, bronchiolitis seems to have a causal effect [51], suggesting respiratory and skin infections play a role in atopy development.

The dysfunctional skin barrier in atopic dermatitis predisposes patients to early infection and allergic sensitization. When allergens are captured and processed by Langerhans cells, the antigen-presenting cells of the epidermis, they migrate to draining lymph nodes and interact with naïve T cells to promote Th2 immunity leading to systemic allergies [66]. Studies show that resultant aeroallergen sensitization is associated with asthma, and one study showed that positive skin prick tests to house dust mite in children 1 or 2 years of age predicted wheeze at age 12 years. Children with AD, wheeze, or both who were sensitized to house dust mite were also at greater risk for wheeze at age 12 than those who were not sensitized, respectively [86]. Findings from the German Multicenter Allergy Study found that allergic rhinitis until the age of 5 years is associated with wheezing between the ages of 5 and 13 years, though this association was not attributable to eczema, perhaps because early allergen sensitization occurred through another mechanism [87]. Interestingly, one study that showed that children with eczema before age 2 years have an increased risk of eczema in preadolescence, but more specifically, those children with eczema in their first year of life but not their second year of life, have a markedly lower risk of eczema in preadolescence than do other children with infantile eczema, in contrast to the view that early onset is associated with worse prognosis, but this group did have increased risks for asthma and rhinitis. That group may represent a particular atopic phenotype, or perhaps the early treatment of eczema altered the risk of subsequent eczema, but not other atopic disease, possibly because early allergic sensitization through the impaired skin barrier already occurred [23]. Findings based on the TLH Study suggested that childhood eczema, especially in association with childhood rhinitis, is strongly associated with atopic asthma in middle age adults that is often still symptomatic. This prospective, population cohort study over 4 decades attributed 20–30% of atopic asthma in adults to a history of childhood eczema and rhinitis, suggesting previous studies on the effects of eczema on adult asthma might have been diluted by considering adult asthma as one condition rather than individual allergic and nonallergic phenotypes [88].

## Role of Filaggrin Mutation in AD and in the Atopic March

Many of the key structural proteins in the outermost layer of the epidermis involved in cornification are encoded for in a locus on chromosome 1q21, which is termed the epidermal differentiation complex (EDC) [89]. Genes found within this locus encode for filaggrin (FLG), a key member of the EDC, in addition to other proteins such as loricrin, involucrin, small proline-rich proteins, late envelope proteins, and the S100 calcium-binding proteins. Discovery of both independent loss-of-function genetic variants (R510× and 2282del4) in the gene encoding filaggrin, whose product is a key structural protein in the outermost layer of the epidermis in up to 50% of patients with AD, provides a greater understanding of the genetic basis for the skin barrier defect in AD [90]. These genetic studies lend strong support to the role of filaggrin in the pathogenesis of AD and in the subsequent progression in the atopic march [91]. The FLG mutations are currently considered a major risk factor for AD, particularly in patients who have onset of AD at 2 years or younger [92]. One study found FLG mutations increased the risk of eczema and food sensitization but not clinical food allergy among 1-year-old infants, suggesting that decreased skin barrier function increases the risk of food sensitization, but other factors may be important in the development of food sensitization to allergy [93]. A longitudinal study evaluated the expected prognosis caused by FLG null mutations among a community-based, physician-diagnosed AD cohort receiving continuous care for almost 5 years, therefore allowing for the waxing and waning nature of atopic dermatitis. Any FLG null mutation was noted in about 16% of the cohort of children, and those children were about 50% less likely than those without mutations to have 6-month time periods with symptom-free skin, with an odds ratio of 0.54. In particular, those with the R501× variant were unlikely to achieve symptom-free skin without topical treatment and were about two times more likely to use topical steroids during any 6-month interval as compared to all others in the cohort [94]. These studies not only demonstrate that FLG mutations increase the risk of eczema and sensitization, but also that clinical outcomes may vary by location of the FLG mutation and influence of other factors.

One study showed a significant association of two filaggrin gene mutations with asthma and allergic rhinitis, but this association was only seen in subjects with the co-existence of AD and was not apparent in subjects without concomitant AD [67]. In addition, filaggrin has not been found to be expressed in the human bronchial epithelium nor beyond the inferior turbinates [95,96]; hence, filaggrin mutations appear not to exert effects in the upper airway, suggesting that the association of filaggrin mutations with other atopic disorders is likely due to the common feature of allergen sensitization through the skin. One study did find an association of FLG mutations with peanut allergy [97]. Although it is possible that some patients with peanut sensitization rather than true clinical allergy were included, this strong association persisted despite multiple variations in peanut allergy diagnostic criteria [98]. The association remained despite asthma status, and the presence of eczema strengthened the association though eczema did not seem to fully account for the association. This study acknowledges that a dysfunctional skin barrier may play a role in the pathogenesis of peanut allergy, and thus supports the theory that sensitization in food allergy occurs in the skin in some patients [97,98]. Peanut protein in household dust is biologically active and related to household peanut consumption, serving as a risk factor for the development of peanut allergy and as a possible nidus for transcutaneous peanut exposure to a young child with AD [99]. It is also possible that cross-sensitization with aeroallergens such as birch protein that share homology with peanut protein play a role in peanut sensitization in AD patients [98]. Oral exposure to allergen, versus transcutaneous exposure, is thought to be more tolerogenic. However, intestinal permeability is increased in some patients with AD. It is unclear if this implies an additional route of allergen penetration. The extent of filaggrin expression in the gastrointestinal tract is currently unknown [97,98].

There is experimental evidence to support the hypothesis that allergen penetration transcutaneously leads to systemic atopic response [100,101]. The fact that asthma is found only in a subset of filaggrin mutation carriers with AD supports the hypotheses that asthma is secondary to allergic sensitization that occurs after epidermal skin barrier impairment. Filaggrin mutations seem likely to play a role in chronicity of disease and IgE sensitization in patients with AD. Studies show that patients with early-onset AD and filaggrin mutations have a tendency to have persistent disease into adulthood [102]. AD patients carrying filaggrin mutations are significantly associated with the extrinsic form of the disease (IgE-mediated sensitization to inhalant or food allergens), and the development of allergic rhinitis and asthma [91,103,104]. The filaggrin mutations predisposing to asthma, allergic rhinitis, and allergic sensitization only in the presence of AD strongly support the role of filaggrin in the pathogenesis of AD and in the subsequent progression along the atopic march. Expression of filaggrin gene is down-regulated in AD skin by Th2 cytokines (IL-4 and IL-13) [105] and normal human keratinocytes by sphingosylphosphorylcholine, which is proinflammatory and proprurigenic in AD [106,107], suggesting that filaggrin defects can develop as an acquired and/or genetic defect. Reduced expression of loricrin and involucrin, two cornified-envelope proteins, have been shown in the lesional skin of AD patients, which contributes to the skin barrier defects in AD [108,109], and their expression was also down-regulated by Th2 cytokines [110].

Experimental evidence for the hypothesis that antigens enter through an impaired epidermal barrier inducing systemic allergen-specific IgE responses is supported in mice with filaggrin frameshift mutation, analogous to human filaggrin mutation. Epicutaneous application of allergen to these mice resulted in cutaneous inflammatory infiltrates and enhanced cutaneous allergen priming with development of IgE antibody responses [100]. Although genetic studies on filaggrin mutation indicate that defective barrier function plays an initial key role in the pathogenesis of AD in many patients, much is still unknown about the sequence of biologic and regulatory events that constitute the transition from an inherited barrier defect to clinical manifestations of eczematous dermatitis and susceptibility to related atopic disorders. The filaggrin gene mutation leads to an epithelial barrier defect and reduced defense mechanisms that allow easy entry for pathogens, allergens and other environmental insults (toxins, irritants, pollutants) followed by polarized Th2 lymphocyte responses with resultant chronic inflammation. However, approximately 40% of carriers of filaggrin gene mutations do not develop AD [111]. Patients with ichthyosis vulgaris, an inherited dry, scaly skin disorder who have filaggrin mutations do not have apparent skin inflammation or infection, which are cardinal features of human AD [112]. Therefore, additional factors may directly and or indirectly interact with filaggrin in the pathogenesis of AD.

## Conclusion

Multiple lines of evidence (clinical, genetic and experimental studies) suggest that previous expression of AD is a prerequisite for the development of allergic rhinitis and asthma and specific sensitization, highlighting the importance of the epidermal barrier in the pathogenesis of these disorders. Whether AD in the atopic march is necessary for progression to other atopic disorders remains to be defined. To establish a causal relationship from AD to airway allergic diseases, evidence of immunological mechanisms accounting for the association and randomized controlled trials demonstrating an effective intervention for AD with reduced subsequent asthma incidence are still needed. It also is important to identify infants at risk for developing lifelong chronic atopic diseases and utilize the critical window of opportunity early in life for therapeutic intervention. Therapy targeting the maintenance and repair of the epidermal barrier in infants with AD may prevent the subsequent development of asthma.
